# A pilot intervention study on bullying prevention among junior high school students in Shantou, China

**DOI:** 10.1186/s12889-022-12669-0

**Published:** 2022-02-09

**Authors:** Zhekuan Peng, Liping Li, Xuefen Su, Yaogui Lu

**Affiliations:** 1 School of Public Health, Shantou University, Shantou, China; 2 Injury Prevention Research Center, Shantou University Medical College, 22 Xinling Road, Shantou, 515041 China; 3 Guangdong Provincial Key Laboratory for Breast Cancer Diagnosis and Treatment, Cancer Hospital of Shantou University Medical College, Shantou, China

**Keywords:** Bullying prevention, Traditional bullying, Cyberbullying, Intervention study

## Abstract

**Background:**

Bullying is common among adolescents and can have an adverse effect on victims. This study aims to implement and evaluate an educational intervention on bullying prevention among junior high school students in Shantou, China.

**Methods:**

The four-session educational intervention was designed based on the knowledge-attitude-practice model and conducted from September to December 2018, with one session implemented a month. The intervention methods included holding bullying-themed class meetings, distributing bullying educational leaflets at school, and playing anti-bullying videos in class. The post-intervention assessment was measured at the end of session 4. The *chi-square* tests were used to examine the changes from baseline to after intervention in the intervention and control group.

**Results:**

The results showed that the intervention group’s awareness of bullying (percentage of the students who knew bullying very well, male: before vs. after intervention: 16.3% vs. 37.6%, *P* < 0.001; female: before vs. after intervention: 11.8% vs. 38.8%, *P* < 0.01), and the female students’ acceptance of anti-bullying education (before vs. after intervention: 89.3% vs. 97.6%, *P* < 0.05) was improved after intervention. The incidence of cyber victimization (male: 32.3% vs. 18.5%, *P* < 0.05; female: 22.4 to 7.0%, *P* < 0.01) was also reduced in the intervention group, with the reduction in the incidence of social (19.4% vs. 8.7%, *P* < 0.05), verbal (40.9% vs. 27.2%, *P* < 0.05) victimization, and peer (36.6% vs. 20.7%, *P* < 0.05) and social bullying (11.8% vs. 2.2%, *P* < 0.01) among male students after intervention.

**Conclusions:**

This educational intervention was the first important step to develop effective intervention strategies for bullying prevention among junior high school students in China.

## Background

Research on bullying can be traced back as early as to the 1970s. Originally, Olweus defined bullying as: When a student is exposed to negative actions, for instance, intentionally inflicts, injury, or discomfort repeatedly and over time on his/her body part, he/she is being bullied [1]. Bullying is an intentional and aggressive behavior and based on an imbalance of power between the perpetrators and the victims [2, 3]. Later on, the definition of traditional bullying was expanded to include four types: physical, social, verbal, and property bullying [4–6]. In recent years, the rapid development of internet and informatization has led to the emergence of cyberbullying, which is different from traditional bullying. Cyberbullying can be defined as repeated aggressive and intentional acts carried out electronically over a period of time by others (a group/an individual), against a victim who cannot defend themselves easily [7].

Worldwide, the prevalence of traditional bullying ranged from 22.8 to 48.2% [8], whereas the prevalence of cyberbullying ranged from 10 to 57% during childhood and adolescence [9–12]. In China, the prevalence of traditional bullying was 26.1% in urban cities [13], and was more prevalent among left-behind children in the rural area, with a prevalence of up to 31.6% [14]. A study conducted in mainland China, Taiwan, and Hong Kong reported a prevalence of 16.7% of cyberbullying and 29.7% of cyber victimization, respectively [15]. As junior high school students are undergoing a critical stage of rapid physical and mental development, both traditional and cyber bullying can have a significant adverse impact on the physical and mental health of adolescent victims [12, 16–18]. These include depression [19], anxiety [20], lowered self-esteem [21] and life satisfaction [22], school absenteeism [23], substance abuse [24], self-harm [25], and even suicide [26].

Considering the widespread prevalence and the serious adverse consequences of bullying among adolescents, it is of essential importance to develop effective intervention strategies, including school-based interventions and anti-bullying legislations, for better prevention and control. A systematic review and meta-analysis reported that school-based anti-bullying programs could decrease traditional bullying by 20 to 23% and victimization by 17 to 20% effectively [27]. Another study not included in the meta-analysis found that a school-wide intervention supporting positive behaviors effectively reduced bullying rate [28]. In addition, one study conducted in the schools of 25 states in the United States indicated that anti-bullying legislation may be effective in reducing students’ risk of being bullied and cyberbullied at school [29].

Despite that more than a quarter of students suffer from bullying, to our knowledge, few anti-bullying and anti-cyberbullying intervention studies have been conducted among Chinese adolescents. Our team conducted a cross-sectional survey investigating bullying prevalence from May to June 2018 in Shantou, a city in the eastern region of Guangdong province in southern China. A total of 3071 junior and senior high school students participated in this cross-sectional study. A high prevalence of bullying was found, with 32.8% students being the victims of bullying by peers and 16.2% having bullied their peers, whereas 24.2% students were cyber bullying victims, and 8.7% had cyberbullied others. In contrast, the awareness of bullying was low: 25.1% students did not know what bullying was, 48.6% students only knew a little bit about bullying, and only 26.3% of them knew bullying very well. In addition, more than half (53.9%) of students had never participated in activities related to bullying prevention, including formal courses or lectures, workshops, or other educational activities.

To address the urgent needs for bullying prevention assessed in the cross-sectional study, our research team designed a pilot intervention study targeting on junior high school students in Shantou. The specific aims were to implement the anti-bullying educational intervention and to test its feasibility and efficacy in changing the students’ knowledge, attitudes, and behaviors regarding bullying, so that to provide a basis for future large-scale interventions. Both traditional bullying and cyberbullying were the targets of the intervention.

## Methods

### Study population

The intervention was conducted from September to December 2018. The three classes in grade 7 of a junior high school participating in the cross-sectional study were selected as the intervention group, and the three classes in the same grade of another school were chosen as the control group. Efforts were made to select the control school similar to the intervention school. Both schools are public schools in the central urban area of Shantou. Most public schools in China follow a 12-year (K-12) educational system, namely, 6 years of primary education (grade 1 - 6) starting at 6 years old, and 6 years of secondary education, including 3 years of junior high school (grade 7 - 9) and 3 years of senior high school (grade 10 -12). As the cross-sectional survey was conducted from May to June 2018, the students were in grade 7 when they participated in the cross-sectional study and promoted to grade 8 when joining in the intervention study. Informed consent was obtained from the school administrator, and all students in the selected classes and their guardians agreed to participate in the study and provided signed consent forms on a voluntary basis. The study was approved by the Ethics Committee of the Shantou University Medical College (SUMC-2018-42).

### Program development

The intervention contents were designed following a rigorous curriculum design procedure. First, extensive literature review was performed to identify existing effective bullying intervention studies. Although several anti-bullying interventions targeting on junior high school students have been conducted internationally, their contents may not be culturally appropriate for the Chinese students. For instance, in the western countries, such as the United States, the socioecological model was widely used to address multiple levels of factors influencing school bullying [30]. The government, non-government organizations, schools, and parents work closely to prevent school bullying. However, considering the current situation of school bullying and bullying prevention in China, the first step we can take is to raise students’ awareness of school bullying through education. After consulting experts in the bullying prevention field in China, we chose the Knowledge-Attitude-Practice (KAP) model as the theoretical framework of the intervention. As a behavioral intervention theory, the KAP model was proposed by G. Cust in the 1960s [31] and has been widely used all over the world in numerous applications in public health. Its fundamental principle was that increasing knowledge will result in changes in attitudes and ultimately lead to changes in practices [32]. A consultation panel of five members, including experts in injury prevention, health promotion and health communication, social medicine, psychology, and epidemiology, was formed. After several rounds of revisions, the contents were finalized and included four major components. According to the KAP model, increasing knowledge is the most important first step, which provides basis for the subsequent changes in attitudes and behavior. Therefore, session 1 focused on introducing the concept and definition of bullying, including the distinction between bullying and appropriate teasing, and the harmful consequences of bullying. Educational leaflets and videos also play important roles in conveying key knowledge and message to the participants through vivid images and animation. Our team designed an educational leaflet and a 20-min video. The leaflets contained information on bullying definition and harmful consequences of being a victim of bullying and the strategies on how to prevent/deal with bullying, whereas the video focused specifically on the potential harm of bullying. Aiming to reinforce the concepts introduced in session 1, we distributed the educational leaflets to the students in session 2, played the video during session 3. Session 4 was also a class meeting, with the theme of emphasizing on the strategies to prevent/deal with bullying. School-bullying statistics in China and examples and symbols of Chinese students were used in all the educational materials. The intervention methods and contents are presented in Table 1. Three facilitators, who were graduate students majoring in Public Health, received standard training before project implementation, and each delivered the four intervention sessions to one class. The four intervention sessions were implemented once a month from September to December 2018. For the control group, educational leaflets with general health information were distributed to the students once a month.

**Table 1:** The intervention methods and contents

Sessions	Intervention time	Intervention methods	Intervention contents
1	September 2018	Bullying-themed class meeting (45 min)	Topics introduced at the class meeting:①the definition of bullying; ②the harmful consequences of bullying
2	October 2018	Distribution of bullying educational leaflets	The leaflets contained the following information: ①the definition of bullying; ②the harmful consequences of bullying; ③ways on how to deal with/ prevent bullying
3	November 2018	Playing videos on bullying (20 min)	The harmful consequences of bullying
4	December 2018	Bullying-themed class meeting (45 min)	Strategies on how to deal with/prevent bullying

### Research design and evaluation

The pre-post intervention research design was adopted to evaluate the efficacy of the intervention program. The pre-test data were extracted from the data of the intervention and control schools in the cross-sectional survey, which was conducted from May to June 2018. The intervention was implemented from September to December 2018, and the post-test was conducted at the end of session 4 in December 2018. In China, an academic year is divided into two terms. The Fall term generally starts on September 1 and ends in January of the following year, whereas the Spring term usually begins in the second half of February and finishes at the end of June. Each term lasts 22 to 23 weeks. Therefore, the time frame assessed in the pre-intervention (May to June 2018) and post-intervention (December 2018) measures was essentially the same, i.e., approximately 4 months.

Both pre- and post-intervention data were collected by using the same questionnaire, which were distributed to the students by the facilitators, and the students completed anonymously. A total of 328 valid questionnaires were received, 184 from the intervention group and 144 from the control group. An ID number was used to match the pre- and post- test questionnaires. A total of 319 students participated in both the cross-sectional study and the intervention study, 178 in the intervention group (consistency rate 96.7%), and 141 in the control group (consistency rate 97.9%).

### Measures

The questionnaire enquired the participants’ sociodemographic characteristics, awareness of bullying, and experience of bullying.

#### Awareness of bullying and acceptance of school anti-bullying education

One question, “*do you know what bullying is”*, was used to assess the students’ awareness of bullying, with the answer options of *don’t know, know a little,* and *know very well*. Another question asked about the students’ willingness to participate in bullying prevention activities, with the answer options of *yes* or *no*.

#### Peer victimization and bullying

The Multidimensional Peer Victimization Scale (MPVS) [4, 5] was used to assess the incidence of victimization during the school term. MPVS was composed of 16 items and included four types of victimization: physical victimization, social victimization, verbal victimization, and property victimization, with each type having 4 items. The Multidimensional Peer Bullying Scale (MPBS) was revised from MPVS by Betts [6], which was used to assess the incidence of bullying during the school term. Similar to MPVS, MPBS also contained 16 items, with four types of bullying: physical bullying, social bullying, verbal bullying, and property bullying. Using the 5-point Likert scoring method, each item had five options – none, 1-2 times per term, 2-3 times per month, once per week, and more than once per week – with each option scored from 0 to 4 and higher scores indicating greater victimization or bullying. If the total score was greater than 1, it indicated victimization or bullying, or a certain type of victimization or bullying. In the cross-sectional survey, the Cronbach’s alpha of MPVS and the four types of victimization were: MPVS: 0.897, physical victimization: 0.810, social victimization: 0.881, verbal victimization: 0.785, and property victimization: 0.773, respectively. The Cronbach’s alpha of MPBS and the four types of bullying were: MPBS: 0.929, physical bullying: 0.882, social bullying: 0.845, verbal bullying: 0.814, and property bullying: 0.889, respectively. The Cronbach’s alpha values suggested that both MPVS and MPBS and the sub-scales had good internal consistency reliability.

#### Cyber victimization and cyberbullying

Cyber victimization and cyberbullying were assessed by using the cyber victimization scale and cyberbullying scale designed by our research team. Both scales included 16 items of negative behaviors, such as insulting, ostracizing, harassing, and rumoring others online. The 5-point Likert scoring method from 0 to 4 was also used, and each item had five options – none, 1-2 times per term, 2-3 times per month, once per week, and more than once per week – with higher scores indicating greater cyber victimization or cyberbullying. A total score greater than 1 indicated cyber victimization or cyberbullying. In the baseline survey, the Cronbach’s alpha of cyber victimization scale was 0.878 and cyberbullying scale 0.947, respectively, also indicating good internal consistency.

### Statistical analysis

A descriptive analysis was performed to describe the participants’ sociodemographic characteristics and the incidence of bullying. *T*-tests and *chi-square* tests were used to examine the changes in awareness, acceptance, and the incidence of bullying from baseline to after the intervention in the intervention group and control group. Due to the significant difference in the gender composition between the intervention and the control group, stratified analysis by gender was performed. *P* values less than 0.05 were considered statistically significant. All the data were analyzed with SPSS Statistics, version 23 (IBM Corporation, Armonk, NY, USA).

## Results

The characteristics of participants in the intervention study are presented in Table 2. After excluding those with missing information on gender, there were 93 (52.2%) male and 85 (47.8%) female students in the intervention group, with an average age of 12.8 ± 0.50 years old, and 56 (39.7%) male and 85 (60.3%) female students in the control group, with the average age of 12.9 ± 0.65 years old. A significant difference was found in gender composition (*P*<0.05), but there was no significant difference in age between the intervention group and the control group (Table 2).

**Table 2:** The characteristics of participants in the intervention study

	Total (***n*** = 319)	Intervention group (***n*** = 178)	Control group (***n*** = 141)	***t***/***χ***^***2***^
Gender, n (%)				
Male	149 (46.7)	93 (52.2)	56 (39.7)	4.963*
Female	170 (53.3)	85 (47.8)	85 (60.3)	
Age (*Mean ± SD*)	12.8 ± 0.57	12.8 ± 0.50	12.9 ± 0.65	1.834
Area of school	–	Urban	Urban	–
Type of school	–	Public	Public	–

*: *P*<0.05; *SD* Standard deviation

### Awareness of bullying and acceptance of school anti-bullying education

Both male and female students in the intervention group increased their awareness of bullying after intervention, and the difference was statistically significant (*P* < 0.05) (Table 3). The percentage of students who did not know bullying decreased from 19.6% before intervention to 7.5% after intervention among boys and reduced from 24.7 to 5.9% among girls. In contrast, the percentage of students who knew bullying very well increased significantly (males: before intervention 16.3% vs. after intervention 37.6%; females: 11.8% vs. 38.8%) (*P* < 0.05). No significant change in the awareness of bullying was found in the control group.

**Table 3:** Students’ awareness of bullying and acceptance of school anti-bullying education

	Male			Female		
	Before intervention n (%)	After intervention n (%)	χ2	Before intervention n (%)	After intervention n (%)	χ2
Awareness of bullying						
Intervention group						
Do not know	18 (19.6) _a_	7 (7.5) _b_		21 (24.7) _a_	5 (5.9) _b_	
Know a little	59 (64.1) _a_	51 (54.9) _a_	13.417**	54 (63.5) _a_	47 (55.3) _a_	22.634***
Know very well	15 (16.3) _a_	35 (37.6) _b_		10 (11.8) _a_	33 (38.8) _b_	
Control group						
Do not know	26 (46.4)	18 (32.2)		22 (25.9)	24 (28.2)	
Know a little	25 (44.7)	32 (57.1)	2.405	48 (56.5)	47 (55.3)	0.132
Know very well	5 (8.9)	6 (10.7)		15 (17.6)	14 (16.5)	
Acceptance of school anti-bullying education						
Intervention group	81 (88.0)	87 (93.5)	1.680	75 (89.3)	83 (97.6)	4.854*
Control group	52 (94.5)	50 (89.3)	1.030	82 (97.6)	80 (94.1)	1.305

*: *P*<0.05, **: *P*<0.01, ***: *P*<0.001

*a* and *b*: different subscripts indicate that the difference between groups is statistically significant

After intervention, 97.6% female students in the intervention group indicated their willingness to participate in educational courses, lectures, or other activities related to bullying prevention, which was significantly higher than 89.3% before intervention (*P* < 0.05) (Table 3). However, the willingness to participate in school-based anti-bullying educational activities did not change significantly among male students in the intervention group or in the control group.

### Incidence of bullying before and after intervention

No significant change was found in the incidence of peer victimization among male or female students in the intervention group. However, social victimization (before intervention: 18 (19.4%) vs. after intervention: 8 (8.7%); *P* < 0.05) and verbal victimization (before intervention: 38 (40.9%) vs. after intervention: 25 (27.2%); *P* < 0.05) was significantly reduced among male students in the intervention group (Table 4).

**Table 4:** The incidence of bullying in the intervention study

The incidence of bullying	Male			Female		
	Before intervention n (%)	After intervention n (%)	χ2	Before intervention n (%)	After intervention n (%)	χ2
Intervention group						
Peer victimization	52 (55.9)	48 (52.2)	0.260	45 (52.9)	40 (46.5)	0.707
Physical victimization	14 (15.1)	13 (14.1)	0.032	1 (1.2)	3 (3.5)	1.000
Social victimization	18 (19.4)	8 (8.7)	4.350*	15 (17.6)	20 (23.3)	0.826
Verbal victimization	38 (40.9)	25 (27.2)	3.858*	27 (31.8)	19 (22.1)	2.034
Property victimization	22 (23.7)	22 (23.9)	0.002	5 (5.9)	7 (8.1)	0.334
Peer bullying	34 (36.6)	19 (20.7)	5.725*	15 (17.6)	9 (10.5)	1.828
Physical bullying	5 (5.4)	4 (4.3)	0.106	0	1 (1.2)	*P* = 0.503
Social bullying	11 (11.8)	2 (2.2)	6.598**	5 (5.9)	4 (4.7)	0.130
Verbal bullying	25 (26.9)	14 (15.2)	3.782	8 (9.4)	4 (4.7)	1.485
Property bullying	2 (2.2)	2 (2.2)	0	0	1 (1.2)	*P* = 0.503
Cyber victimization	30 (32.3)	17 (18.5)	4.634*	19 (22.4)	6 (7.0)	8.097**
Cyberbullying	8 (8.6)	3 (3.3)	2.359	5 (5.9)	1 (1.2)	2.812
Control group						
Peer victimization	11 (19.6)	9 (16.1)	0.243	18 (21.2)	19 (22.4)	0.035
Physical victimization	1 (1.8)	2 (3.6)	0.343	0	0	–
Social victimization	5 (8.9)	1 (1.8)	2.818	5 (5.9)	10 (11.8)	1.828
Verbal victimization	7 (12.5)	4 (7.1)	0.907	11 (12.9)	13 (15.3)	0.194
Property victimization	2 (3.6)	2 (3.6)	0	7 (8.2)	3 (3.5)	1.700
Peer bullying	7 (12.5)	4 (7.1)	0.907	6 (7.1)	2 (2.4)	2.099
Physical bullying	1 (1.8)	1 (1.8)	0	0	0	–
Social bullying	0	0	–	0	0	–
Verbal bullying	5 (8.9)	3 (5.4)	0.538	5 (5.9)	1 (1.2)	2.764
Property bullying	0	0	–	0	1 (1.2)	*P* = 0.500
Cyber victimization	9 (16.1)	4 (7.1)	2.176	19 (22.4)	15 (17.6)	0.588
Cyberbullying	1 (1.8)	0	*P* = 0.500	4 (4.7)	3 (3.5)	0.149

*: *P*<0.05, **: *P*<0.01, ***: *P*<0.001; The *P* values of the Fisher’s exact tests were reported in the table directly

In terms of peer bullying, the incidence was significantly decreased from 34 (36.6%) to 19 (20.7%) (*P* < 0.05) after intervention among male students. However, the reduction was not significant among female students in the intervention group. For the different types of peer bullying, a significant reduction was only observed for social bullying among male students in the intervention group (before intervention: 11 (11.8%) vs. after intervention: 2 (2.2%); *P* < 0.01).

The incidence of cyber victimization decreased significantly among both male (before intervention: 30 (32.3%) vs. after intervention: 17 (18.5%); *P* < 0.05) and female (before intervention: 19 (22.4%) vs. after intervention: 6 (7.0%); *P* < 0.01) students in the intervention group, but no significant reduction was found for cyberbullying.

No significant change in the incidence of peer or cyber victimization or bullying was observed in the control group.

## Discussion

We observed increased awareness and reduced incidence of cyber victimization among junior high school students after intervention in urban Shantou, China. However, gender difference was observed. Whereas reductions in social and verbal victimization, peer bullying, and social bullying were found only among male students, an increase in the acceptance of school-based anti-bullying education was observed only among female students in the intervention group.

According to the KAP model, improving students’ awareness and knowledge of bullying is the first important step to reduce and prevent the incidence of bullying. Many students and even their parents or teachers cannot distinguish the difference between bullying and appropriate teasing [33]. The results of this study show that conveying information and knowledge through education to help students to understand the difference is very important and effective to improve their awareness. However, despite a higher incidence of both peer and cyber victimization and bully, boys were less interested in participating in anti-bullying educational activities than girls. This was in line with the findings in a previous study which indicated that girls showed stronger anti-bullying attitudes than boys [34]. The likely explanation may be that male students are probably not as sensitive to and have not yet recognized the harmful effect of bullying, especially psychological bullying, on their physical and mental health, as female students.

Interestingly, although male students were less likely to show interest in participating anti-bullying education, the reductions in the incidence of victimization, particularly social and verbal victimization, and peer bullying, especially social bullying, were mainly observed among male students. Similarly, a meta-analysis also found that bullying prevention programs were more effective in reducing bullying incidence among boys than girls [35]. One potential explanation is that bullying among female students tends to occur in the friend groups more often than among male students [36]. Before the intervention, girls may not realize that they are bullying or being bullied by their friends. However, after the intervention, with increased awareness they did realize the occurrence of bullying, thus canceling out the effects of intervention. In addition, friendships are considered extremely important for adolescents, especially among female students [37]. Although girls may realize that they are being bullied by a friend, they may be reluctant to report the bullying incidents, because they do not want to lose their friend(s). Another possible explanation could be due to the relatively higher baseline incidence of social and verbal victimization and peer bullying, including social bullying, and lower physical and property victimization and bullying among male students at baseline, and the much lower incidence of physical and property bullying among female students. Once the male students’ changed their awareness and realized that these were negative behaviors through participating in the intervention, they were likely to change such types of negative behaviors. Similarly, the incidence of cyber victimization was relatively high among female students before the intervention, and a significant reduction was also observed in this group.

This study has several limitations. First, as this was a pilot study, we only selected three classes in grade 8 at the time of intervention from two schools in the urban district but did not include students in other grades or in the suburban district or island county in Shantou. The sample size is small. Although we tried to select two similar schools (both are public schools located in the urban district), differences in bullying-related awareness, acceptance, and incidence were found at baseline, and the rates of bullying and victimization were lower in the control group than the intervention group, except for cyberbullying. Therefore, the observed changes cannot be solely attributed to the intervention itself. In addition, the results may not be generalizable to other high school students beyond the study population. Based on the pilot results, we will revise the program and expand the intervention to cover more students in other grades and regions. Second, the intervention methods were simple and mainly focused on education. The duration of educational intervention on bullying was short, therefore, some intervention effects may not be obviously observed. A “dose response” relationship was found between bullying interventions and the intervention effect, and the intensity and duration of the intervention was directly linked to its effectiveness in previous studies [27, 38]. To have greater effects, more intensive intervention with longer duration should be designed and implemented. The KAP model, the main theoretical framework used in the intervention, may not be sufficient to induce bullying-related behavioral change. Considering the effectiveness of whole-school anti-bullying approaches demonstrated in previous studies [1], we will adopt such strategies and incorporate whole-school elements when revising the intervention methods to address multiple levels of factors influencing student bullying. Furthermore, due to limited time and funding, no follow-up was conducted. Whether the increased awareness and reduced incidence observed in the intervention group can be sustained beyond the intervention period remain unclear. Thirdly, the students’ roles in bullying were divided as victims and perpetrators in our intervention. However, in real school life, some students can be both victims and perpetrators at the same time. How the intervention can impact on this group of students is not clear in the current study.

Despite these limitations, the intervention was well designed and implemented, and positive changes in bullying-related awareness and incidence were observed in the intervention group. The results of our study were consistent with the findings from previous school-based interventions conducted in the western countries and provided the first valuable evidence of the efficacy of bullying intervention in China.

Based on the socioecological model, school bullying is a complicated behavior and phenomenon that is influenced by multiple levels of factors. When designing school-based anti-bullying interventions, researchers need to take these factors, including individual, family, school, community, and societal factors, into account. Considering the effectiveness of anti-bullying policies found in the United States [29], researchers need to collaborate closely with school principals and senior leaders to create and promote an anti-bullying school policy and atmosphere and strengthen the disciplinary actions against bullying. Schools should implement “zero-tolerance” policy towards bullying and create an anti-bullying school culture. It is also necessary for policymakers to develop policies to protect victims and discipline perpetrators. Parents should also be actively involved in the anti-bullying efforts and work closely with schools to fight against different types of bullying effectively. For instance, school-parent meetings with the theme of anti-bullying should be held on a regular basis to increase parents’ awareness of bullying [39].

## Conclusion

An anti-bullying educational intervention was designed and pilot-tested among junior high school students in Shantou, China. The preliminary results showed positive changes in bullying-related awareness, willingness to participate in bullying prevention activities, and incidence of cyber victimization and traditional peer bullying and victimization after intervention, and the results differed by gender. The current intervention laid down the first important step in the anti-bullying efforts at schools in China. A whole-school intervention approach needs to be adopted in future school bullying prevention programs.

## Data Availability

The datasets of this study is available from the corresponding author on reasonable request.

## Abbreviations

KAP: Knowledge-Attitude-Practice
MPVS: The Multidimensional Peer Victimization Scale
MPBS: The Multidimensional Peer Bullying Scale
SD: Standard Deviation

## References

[1] Olweus D (1994). Bullying at school: basic facts and effects of a school based intervention program. J Child Psychol Psychiatry.

[2] Vaillancourt T, Mcdougall P, Hymel S, Krygsman A, Miller JL, Stiver K (2008). Bullying: are researchers and children/youth talking about the same thing?. Int J Behav Dev.

[3] Gladden RM, Vivolo-Kantor AM, Hamburger M, Lumpkin C (2014). Bullying surveillance among youths: uniform definitions for public health and recommended data elements, Version 1.0. Centers for Disease Control & Prevention.

[4] Mynard H, Joseph S (2000). Development of the multidimensional peer-victimization scale. Aggress Behav.

[5] Raine A, Fung AL-c, Lam BYH. (2011). Peer victimization partially mediates the schizotypy-aggression relationship in children and adolescents. Schizophr Bull.

[6] Betts LR, Houston JE, Steer OL (2015). Development of the multidimensional peer victimization scale–revised (MPVS-R) and the multidimensional peer bullying scale (MPVS-RB). J Genet Psychol.

[7] Smith PK, Mahdavi J, Carvalho M, Fisher S, Russell S, Tippett N (2008). Cyberbullying: its nature and impact in secondary school pupils. J Child Psychol Psychiatry.

[8] Gaffney H, Farrington DP, Ttofi MM. Examining the Effectiveness of School-Bullying Intervention Programs Globally: a Meta-analysis. Int J Bullying Prev. 2019;1:14–31.

[9] Callaghan M, Kelly C, Molcho M (2015). Exploring traditional and cyberbullying among Irish adolescents. In J Public Health.

[10] Zhou ZK, Tang HY, Tian Y, Wei H, Zhang FJ, Morrison CM (2013). Cyberbullying and its risk factors among Chinese high school students. Sch Psychol Int.

[11] Rice E, Petering R, Rhoades H, Winetrobe H, Goldbach J, Plant A (2015). Cyberbullying perpetration and victimization among middle-school students. Am J Public Health.

[12] Sourander A, Brunstein Klomek A, Ikonen M, Lindroos J, Luntamo T, Koskelainen M (2010). Psychosocial risk factors associated with cyberbullying among adolescents: a population-based study. Arch Gen Psychiatry.

[13] Han Z, Zhang G, Zhang H. School Bullying in Urban China: Prevalence and Correlation with School Climate. Int J Environ Res Pub Health. 2017;14(10):1116. 10.3390/ijerph14101116 PMC5664617 28946682

[14] Zhang H, Zhou H, Cao R. Bullying victimization among left-behind children in rural China: Prevalence and associated risk factors. J Interpersonal Violence. 2021;36(15-16):NP8414–NP8430. 10.1177/0886260519843287 30983481

[15] Chen J-K, Chen L-M (2020). Cyberbullying among adolescents in Taiwan, Hong Kong, and mainland China: a cross-national study in Chinese societies. Asia Pac J Soc Work Dev.

[16] Wolke D, Lereya ST (2015). Long-term effects of bullying. Arch Dis Child.

[17] Fong-Ching C, Ching-Mei L, Chiung-Hui C, Wen-Yun H, Tzu-Fu H, Yun-Chieh P (2013). Relationships among cyberbullying, school bullying, and mental health in Taiwanese adolescents. J Sch Health.

[18] Landstedt E, Persson S (2014). Bullying, cyberbullying, and mental health in young people. Scand J Public Health.

[19] Selkie EM, Kota R, Chan Y-F, Moreno M (2015). Cyberbullying, depression, and problem alcohol use in female college students: a multisite study. Cyberpsychol Behav Soc Netw.

[20] Ford R, King T, Priest N, Kavanagh A (2017). Bullying and mental health and suicidal behaviour among 14- to 15-year-olds in a representative sample of Australian children. Aust New Zealand J Psychiatry.

[21] Wang X, Zhang Y, Hui Z, Bai W, Terry P, Ma M (2018). The mediating effect of regulatory emotional self-efficacy on the association between self-esteem and school bullying in middle school students: a cross-sectional study. Int J Environ Res Public Health.

[22] Varela JJ, Guzmán J, Alfaro J, Reyes F (2019). Bullying, Cyberbullying, student life satisfaction and the Community of Chilean Adolescents. Appl Res Qual Life.

[23] Hinduja S, Patchin JW (2008). Cyberbullying: an exploratory analysis of factors related to offending and victimization. Deviant Behav.

[24] Calvete E, Orue I, Gámez-Guadix M (2016). Cyberbullying victimization and depression in adolescents: the mediating role of body image and cognitive schemas in a one-year prospective study. Eur J Crim Policy Res.

[25] Esposito C, Bacchini D, Affuso G (2019). Adolescent non-suicidal self-injury and its relationships with school bullying and peer rejection. Psychiatry Res.

[26] Peng Z, Klomek AB, Li L, Su X, Sillanmäki L, Chudal R (2019). Associations between Chinese adolescents subjected to traditional and cyber bullying and suicidal ideation, self-harm and suicide attempts. BMC Psychiatry.

[27] Ttofi MM, Farrington DP (2011). Effectiveness of school-based programs to reduce bullying: a systematic and meta-analytic review. J Exp Criminol.

[28] Waasdorp TE, Bradshaw CP, Leaf PJ (2012). The impact of Schoolwide positive behavioral interventions and supports on bullying and peer rejection: a randomized controlled effectiveness trial. Arch Pediatr Adolesc Med.

[29] Hatzenbuehler ML, Schwab-Reese L, Ranapurwala SI, Hertz MF, Ramirez MR (2015). Associations between Antibullying policies and bullying in 25 states. JAMA Pediatr.

[30] Swearer SM, Espelage DL (2003). Introduction: A social-ecological framework of bullying among youth. Bullying in American schools: A Social-Ecological Perspective on Prevention and Intervention.

[31] Cust G (1966). Why health education?. Dist Nurs.

[32] Sambo M, Lembo T, Cleaveland S, Ferguson HM, Sikana L, Simon C (2014). Knowledge, attitudes and practices (KAP) about rabies prevention and control: a community survey in Tanzania. PLoS Negl Trop Dis.

[33] Mills CB, Carwile AM. The good, the bad, and the borderline: Separating teasing from bullying. Commun Educ. 2009;58(2):276–301.

[34] Salmivalli C, Kaukiainen A, Voeten M (2005). Anti-bullying intervention: implementation and outcome. Br J Educ Psychol.

[35] Kennedy RS. Gender differences in outcomes of bullying prevention programs: A meta-analysis. Child Youth Serv Rev. 2020;119:105506.

[36] Mishima K (2003). Bullying amongst close friends in elementary school. Japan J Soc Psychol.

[37] Berndt TJ (1982). The features and effects of friendship in early adolescence. Child Dev.

[38] Olweus D (2005). A useful evaluation design, and effects of the Olweus bullying prevention program. Psychol Crime Law.

[39] Fekkes M, Pijpers FIM, Verloovevanhorick SP (2004). Bullying: who does what, when and where? Involvement of children, teachers and parents in bullying behavior. Health Educ Res.

